# Imaging of pericardial tumours: a case report

**DOI:** 10.1186/1476-7120-4-29

**Published:** 2006-07-18

**Authors:** Lindsey Tilling, Lucy Hudsmith, Jonathan Goldman, Harald Becher

**Affiliations:** 1 The John Radcliffe Hospital, Oxford, UK

## Abstract

**Background:**

Pericardial tumours are unusual and may be difficult to characterise with imaging. They manifest as large, non-contractile, solid masses within the pericardium. Presenting symptoms include heart failure, arrythmias, sudden death, cyanosis and chest pain.

**Case presentation:**

We describe a case of massive pericardial fibroma in a 52 year old woman, who presented with palpitations only.

**Conclusion:**

We illustrate the different imaging modalities available to image this tumour prior to surgical resection, and indicate the strengths and weaknesses of each.

## Background

Pericardial tumours are infrequently encountered in clinical practice; one study suggests an incidence of between 2–3% of all primary cardiac tumours [1]. They may present with non-specific symptoms such as heart failure, arrhythmias, sudden death, cyanosis, and chest pain. Imaging can be challenging; echocardiography, computerized tomography and magnetic resonance imaging may all be employed. Accurate assessment is necessary to define the location, tissue composition and benign or malignant nature of the mass, which carries significant clinical implications during surgical resection.

## Case presentation

**Figure 1:**
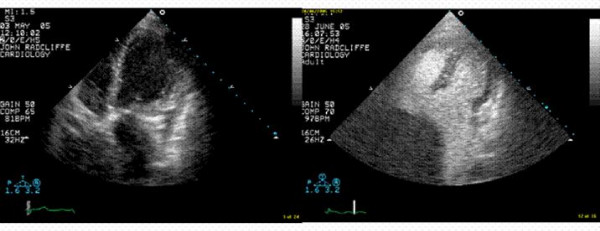
Transthoracic 4 chamber echocardiogram demonstrating noisy native recording with poor visualization of the right atrium. **B **Transthoracic 4 chamber contrast echocardiogram demonstrating large mass superior to right atrium.

A 52 year old lady presented to her general practitioner with palpitations. She had no significant previous medical history, and was taking no medications. Examination was unremarkable. The electrocardiogram showed sinus rhythm. Echocardiography gave a noisy recording but revealed a moderate sized pericardial effusion without haemodynamic compromise, dilated left atrium, and a suspicion of a right atrial mass. (Figure 1a)

Chest x-ray revealed a grossly enlarged asymmetrical heart (cardiothoracic ratio 24.4/31.3), with enlargement of the right atrium. The appearances were thought to be in keeping with a tumour of pericardial or cardiac origin.

A contrast echocardiogram was performed; Sonovue contrast given for endocardial border definition revealed a 12 cm vascular mass superior to the right atrium, to the right of the sternum, likely to be attached to the pericardium. (Figure 1b/Additional File 1).

**Figure 2:**
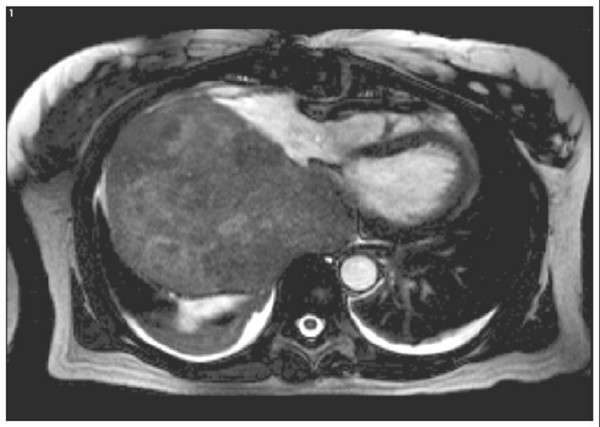
Magnetic resonance image demonstrating huge right sided mass within the chest.

Further imaging was undertaken, to define the location of the mass. A computerised tomography of the chest found a massive, heterogeneously enhancing mass outside the right atrium, directly abutting the right anterior pleural surface. The right inferior pulmonary vein was partly compressed. Magnetic resonance imaging of the chest however, suggested the mass was of intra atrial origin, as the right atrium was seen to have a concave interface with the mass, and there was distension of the atrial septum to the left. It was thought to be a myxoma (Figure 2).

**Figure 3:**
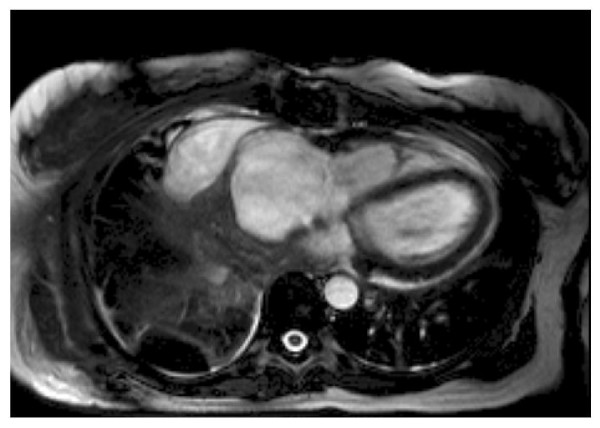
Magnetic resonance image obtained during post-operative surveillance, showing no recurrence of the tumour.

The mass was surgically resected. It measured 150 × 115 × 90 mm and weighed 830 grammes. It was found to be intrapericardial, lying in the right posterolateral pericardial space, compressing and deforming the right atrium. Histology revealed a benign fibrous tumour. The patient was discharged a week after surgery and has not experienced any further symptoms. Subsequent MR imaging shows no recurrence of the tumour (Figure 3).

## Discussion

We have presented a case of a giant pericardial tumour identified by echocardiography, further defined by contrast. This case illustrates an unusual tumour presenting with a common symptom. A 25 year retrospective study found only 3 of 125 primary cardiac and pericardial tumours were fibromas [1]. An audit of 23 patients with cardiac fibroma found the presenting symptoms included heart failure, arrhythmias, sudden death, cyanosis, and chest pain [2].

We also demonstrate the different imaging modalities available in preoperative assessment of cardiac masses, and that they do not always concur. Cardiac fibromas manifest as a large, noncontractile, solid mass in a cavity-enclosing structure (ventricular wall, pericardium etc) at echocardiography, and as a homogeneous mass with soft-tissue attenuation at CT. They are usually homogeneous and hypointense on T2-weighted MR images and isointense relative to muscle on T1-weighted images. Magnetic resonance is well established as the imaging modality of choice for assessment of suspected cardiac mass, and can prove useful for tissue characterization and differentiation between benign and malignant structures [3].

## Conclusion

In this particular case, echocardiography identified the mass, chest x-ray confirmed its presence, and CT correctly identified the mass to be outside the right atrium, but suggested it was of pleural origin. MR failed to identify the location and tissue composition of the tumour, but correctly identified a benign lesion. We conclude that several imaging techniques are required for optimal preoperative assessment. Our suggestions for clinical practice are: 1) clear visualisation of the right heart is necessary in any transthoracic echo study. If this is not possible, contrast should be used. 2) x-ray imaging, though not specific for cardiac lesions, can help confirm a suspicion of existence. 3) Advanced imaging should be utilised pre-operatively, but final diagnosis is often only possible following resection.

## Competing interests

The author(s) declare that they have no competing interests.

## Authors' contributions

All authors were involved in imaging. LT drafted the manuscript. All authors read and approved the final manuscript.

## Supplementary Material

Additional File 1 Transthoracic 4 chamber contrast echocardiogram demonstrating large mass superior to right atrium. Click here for file
